# Implementation of helicase-dependent amplification with SYBR Green I for prompt naked-eye detection of bacterial contaminants in platelet products

**DOI:** 10.1038/s41598-023-30410-8

**Published:** 2023-02-24

**Authors:** Warangkana Yamket, Panuwat Sathianpitayakul, Pitak Santanirand, Panan Ratthawongjirakul

**Affiliations:** 1 Program of Molecular Sciences in Medical Microbiology and Immunology, Faculty of Allied Health Sciences, Chulalongkorn University, Bangkok, 10330 Thailand; 2 Microbiology Unit, Faculty of Medicine Ramathibodi Hospital, Mahidol University, Bangkok, 10400 Thailand; 3 Research Unit of Innovative Diagnosis of Antimicrobial Resistance, Faculty of Allied Health Sciences, Chulalongkorn University, Bangkok, 10330 Thailand

**Keywords:** Health care, Medical research

## Abstract

Platelet transfusions may lead to more significant risks of infection and septic transfusion reactions that can be fatal to the recipient. Platelet products should be screened to limit or detect bacterial contamination before application to patients to minimise any adverse reactions. This study aimed to develop a helicase-dependent amplification (HDA) technique targeting a universal highly conserved bacterial gene, 16S rRNA, in combination with naked-eye detection using SYBR Green I (HDA/SYBR Green I) to detect bacterial contamination in platelet products. Thirty positive samples were obtained from spiked platelet products by five transfusion-relevant bacterial strains and were screened for bacterial contamination by HDA/SYBR Green I. HDA/SYBR Green I showed an enhanced yield of bacterial contaminant detection when performed with medium to late shelf life, Day 2 of storage or later platelet products (98.67% sensitivity and 100% specificity compared to the BACT/ALERT culture system). The limit of detection of HDA/SYBR Green I was 1 ng, and there was no cross-reaction with other organisms that could likely contaminate platelet products. The developed HDA/SYBR Green I assay is rapid and simplistic and only requires an easy-to-find heat box, available in general blood bank laboratories, for the amplification step. This technique is suitable for further development as an alternative method to detect bacterial contamination in platelet products in the near future.

## Introduction

Bacterial contamination of blood components is a longstanding concern^1^. Receiving bacterial contaminated blood components may lead to transfusion-transmitted fatalities due to bacterial infections. The U.S. Food and Drug Administration (FDA) report from 2012 to 2016 showed that transfusion-transmitted bacterial infections were the third cause of death from transfusions after transfusion-related acute lung injury (TRALI) and transfusion-associated circulatory overload (TACO)^2^. Even the blood collection process is being improved and is well-controlled to reduce the bacterial contamination of blood components^3^. However, platelet products present a significant risk due to their storage conditions (20–24 °C with agitation), which may support bacteria to multiply and reach clinically harmful levels during their 5-day storage period^1^.

Regarding the American Association of Blood Banks (AABB) standard 5.1.5.1, platelet products should be screened to limit or detect bacterial contamination before application to patients to minimise any adverse reactions^4^. Bacterial culture is the gold standard for detecting bacterial contamination from various blood components. However, the traditional culture exhibits some limitations, including an inappropriate practice for bacterial culture in the blood bank working area, time consumption that does not match the short 5-day shelf life of platelets, and high cost-effectiveness of the assay. The BacT/ALERT® culture system is an automated culture-based method for platelet quality control approved by the FDA. Several molecular-based methods have revolutionised the detection of bacterial contamination in platelet products, such as PCR and real-time PCR. However, these techniques are not widely used in routine testing due to a complicated process, high cost-effectiveness, and specific instrument dependence that requires ongoing maintenance^1,5^.

The isothermal amplification method is an amplification technique that operates under a constant temperature. The most significant advantage of isothermal amplification is that it does not require a thermocycling machine. Therefore, isothermal amplification requires low energy consumption and can be easily integrated into a simple detection platform, such as in the field or instrumentation-free settings. Helicase-dependent amplification (HDA) is one of the most straightforward approaches for isothermal nucleic acid amplification, mimicking an in vivo process of DNA replication, using a helicase instead of heat to isothermally unwind DNA duplexes. The process occurs under an optimum temperature range from 60 to 65 °C and takes 30–120 min. HDA has been previously developed as a novel molecular-based tool for rapid pathogen detection^6,7^. The aim of this study was to fabricate a simple, rapid, and sensitive screening method for bacterial contaminants in platelet products that can be easily implemented in blood bank service routines. To achieve this objective, HDA amplification targeting 16S rRNA was used with endpoint detection using SYBR Green I (referred to as HDA/SYBR Green I).

## Materials and methods

### Representative bacterial strains

Five strains of bacteria, including *Staphylococcus aureus* (ATCC 29,523), *Staphylococcus epidermidis*, *Bacillus cereus*, *Escherichia coli* (ATCC 25,922), and *Serratia marcescens*, were used as representative strains. All of these strains have been previously reported as common transfusion-relevant bacterial strains of platelet products^1,8,9^. The ATCC bacterial strains were kindly provided by Assoc. Prof. Dr. Pitak Santanirand, the Microbiology Unit, Faculty of Medicine Ramathibodi Hospital, Mahidol University, Bangkok, Thailand. *S. epidermidis*, *B. cereus*, and *S. marcescens* were clinical isolates obtained from leftover specimens from the Microbiology Unit, Faculty of Medicine Ramathibodi Hospital, Mahidol University, Bangkok, Thailand, and were confirmed at the species level by conventional biochemical tests and PCR sequencing. The bacteria were subcultured on tryptic soy agar (TSA) at 37 °C for 24 h before use. Bacterial genomic DNA was extracted by the boiling method. A single 24 h colony of each strain was resuspended in 1 ml of sterile distilled water and boiled at 95 °C for 10 min, followed by a 5-min centrifugation at 12,000 rpm. The supernatant was used as a DNA template for HDA/SYBR Green I optimisation.

### Primer design and screening

An HDA/SYBR Green I assay was developed targeting the highly conserved bacterial 16S rRNA gene, the most widely used marker gene employed to identify bacteria and differentiate between closely related bacterial species. HDA primers were designed from the conserved fragments of 16S rDNA sequences predicted to cover 275,057 eubacterial species derived from the Ribosomal Database Project of a previous study^10^ using the Primer3 program (version 0.4.0; http://bioinfo.ut.ee/primer3-0.4.0/). This selected 16S rRNA gene fragment is dominant among Firmicutes, Gemmatimonadetes and Proteobacteria, which are the predominant phyla in all microbiomes^10^. These HDA primer candidates contained 20–32 bases, as recommended by the IsoAmp®II Universal tHDA kit (New England BioLabs, USA). The primer melting temperature ranged from 60 to 74 °C, and the GC% was within the interval of 35–60% ^6^. The OligoAnalyser 3.1 program (https://eu.idtdna.com/calc/analyser) and BLAST software (https://blast.ncbi.nlm.nih.gov/Blast.cgi) were used to assess the possibility of secondary structure formation and the specificity of the primers, respectively. Four forward and reverse primers were designed in this study. Each forward and reverse primer candidate was paired and preliminarily screened for its ability to amplify specific targets with an IsoAmp®II Universal tHDA kit as recommended by the manufacturer. The most suitable primer pair was selected and finally used in our fabricated HDA/SYBR assay as follows: 16S rRNA forward (5’AGTCCCRYAACGAGCGCAACCC 3’) and 16S rRNA reverse (5’TTGACGTCRTCCCCRCCTTCC 3’), generating an HDA product size of 104 bp.

### HDA/SYBR green I optimisation

The optimised HDA/SYBR Green I assay was evaluated using genomic DNA of *S. aureus* (ATCC 29,523), *S. epidermidis*, *B. cereus*, *E. coli* (ATCC 25,922), and *S. marcescens* as positive controls to obtain the most appropriate conditions for HDA amplification and endpoint SYBR Green I detection. During HDA/SYBR Green I assay development, the amount of DNA template (1–20 ng), primer concentration (50–200 nM), MgSO_4_ concentration (3–4.5 mM), NaCl concentration (20–50 mM), incubation temperature (63–69 °C in the heating block) and incubation period (15–90 min) were optimised until the most appropriate conditions were obtained. Sterile distilled water served as a negative control. Amplified 16S rRNA was directly detected by the naked-eye in natural light by adding 1 µL of 100–500 × SYBR to a final volume of 50 µL of HDA products and immediately observing a colour change of the solution. The solution changed from light orange to bright green in the presence of the HDA product, indicating that bacterial DNA was detected. Conversely, the solution remained light orange in the absence of the HDA product, suggesting that no bacterial DNA was detected.

In every optimisation step, the HDA products were examined by SYBR Green I and compared with agarose gel electrophoresis. The condition giving clear readout results when observed with SYBR Green I, without nonspecific bands upon agarose gel electrophoresis, was chosen (data not shown).

### Spiked platelet product samples for the performance test

Spiked platelet samples were established to mimic natural bacterial-contaminated platelet products. This study used sterile leukocyte-poor platelet concentrates (LPPC) supplemented with a platelet additive solution (PAS). All the platelet concentrates were due to the expiration of the shelf life (after Day 5 of storage) and were collected from the Regional Blood Centre IV, Thai Red Cross Society. The discarded platelet products were checked for purity by plating out onto blood agar and observed for any growth colony until 7 days of aerobic and anaerobic incubations. The study was approved by the Research Ethics Committee, National Blood Centre, Thai Red Cross Society, Bangkok, Thailand (COA.NBC 15/2019).

Five strains of bacteria, including *S. aureus* (ATCC 29,523), *S. epidermidis*, *B. cereus*, *E. coli* (ATCC 25,922), and *S. marcescens*, were grown overnight in tryptic soy broth (TSB) and adjusted to 0.5 McFarland standard (10^8^CFU/ml). Serial tenfold dilutions were prepared in TSB to achieve a concentration of 10^2^ CFU/ml. Then, 3 ml of each bacterial strain was injected into a separate bag containing 297 ml of sterile platelet products, achieving a final bacteria concentration of 1 CFU/ml (One strain per bag). The freshly prepared spiked sample was named Day 0 (representing the collection day). The platelet samples were maintained under standard platelet storage conditions at 20–24 °C under agitation until 5 days and named Day 1 to Day 5, respectively. During storage, platelet products were sampled daily from every bag (Day 0–5) inoculated with each bacterial strain. In total, 30 contaminated samples were obtained and set as the positive samples. Twelve sterile samples were set as the negative samples. Bacterial DNA was extracted from 1 ml of daily aliquoted platelet products using the QIAamp DNA Microbiome Kit (Qiagen, Germany) as recommended by the manufacturer. The DNA was stored at −20 °C until use. All experiments were performed in 5 separate replicates. Another 1 ml of platelet product was aliquoted daily, and tenfold dilutions were prepared in TSB and plated on TSA. Colony counting was performed after 24 h of incubation to determine the bacterial concentration of each strain in the platelet product on each storage day. The experiments were performed in triplicate.

### BacT/ALERT® culture system protocol

A 10 ml aliquoted sample was taken from Day 0 to Day 5 platelet products and inoculated into BacT/ALERT® BPA culture bottles (BioMérieux, France). The samples were incubated with the automated BACT/ALERT system for 7 days, and CO_2_ production indicating bacterial growth was automatically measured via a fluorescent signal. All positive bottles were subcultured, and bacterial colonies were reidentified for confirmation by the matrix-assisted laser desorption/ionization (MALDI-TOF) Microflex series (Bruker Diagnostics, Germany).

### HDA/SYBR green I assay protocol

**Figure 1:**
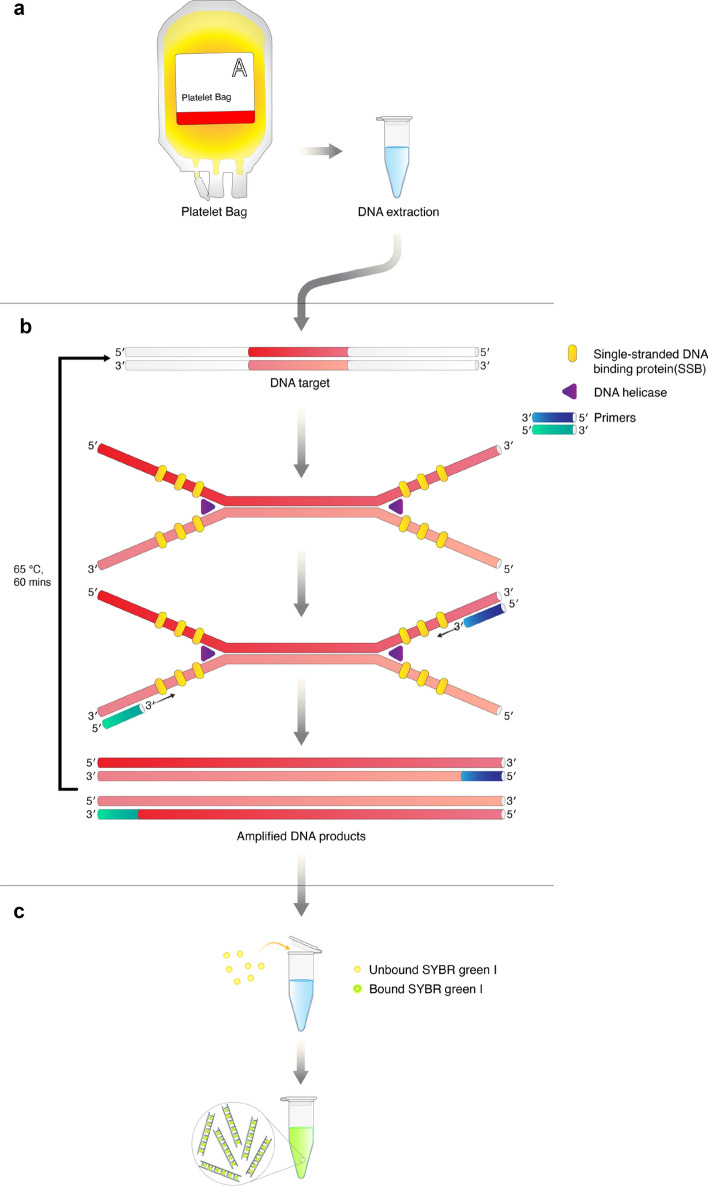
The diagram presentation of the process and reaction principle of HDA/SYBR Green I. (**a**) DNA extraction. One millilitre of daily aliquoted platelet products was extracted using the QIAamp DNA Microbiome Kit. (**b**) HDA reaction. Ten nanograms of DNA were used as a template in the HDA reaction under 65 °C for 60 min. (**c**) SYBR Green I detection. One microlitre of 400 × SYBR Green I was added to HDA products. A colour change was observed by the naked-eye.

DNA samples derived from Day 0 to Day 5 of platelet products containing 5 individual bacterial strains were used in the HDA/SYBR Green I assay protocol as positive contaminated samples. In contrast, sterile distilled water and sterile platelet product were used as negative samples. The HDA reaction was performed with the IsoAmp®II Universal tHDA kit (New England BioLabs, USA) as recommended by the manufacturer. Each HDA reaction consisted of 5 µM 16S rRNA gene forward and reverse primers, 5 µl 10X annealing buffer II, 2 µl MgSO_4_, 4 µl NaCl, 3.5 µl IsoAmp dNTPs solution, 3.5 µl IsoAmp enzyme mix, 10 ng of DNA and sterile distilled water up to 50 µl. The HDA reaction mixture was incubated at 65 °C for 60 min in the heating block. After amplification, each reaction was aliquot into two separate tubes for evaluation by different detection methods. The first tube was evaluated by SYBR Green I (HDA/SYBR Green I assay), in which 1 µl of 400 × SYBR Green I (Takara Bio, Japan) was directly added to the 25 µl HDA products. A colour change of the solution in natural light was immediately observed by the naked-eye. The second tube was evaluated by 2% agarose gel electrophoresis. Without prior purification, 2 µl of HDA product was directly mixed with 1 µl of a loading dye before loading into 2% agarose gel electrophoresis. Randomly selected HDA products were confirmed by Sanger DNA sequencing. The sequence reads were compared to gene sequences described in the NCBI database (Blast search http://blast.ncbi.nlm.nih.gov/Blast.cgi). All experiments were performed in 5 replicates. HDA/SYBR Green I was blindly interpreted by two different investigators to eliminate bias. The principles underlying HDA/SYBR Green I assay for the detection of bacterial contamination platelet products are illustrated in Fig. 1.

### Analytical limit of detection

An analytical limit of detection was established to determine the lowest concentration of DNA enabling detection by the HDA/SYBR Green I assay. Genomic colony DNA of *S. aureus* (ATCC 29,523), *S. epidermidis*, *B. cereus*, *E. coli* (ATCC 25,922), and *S. marcescens* was tenfold serially diluted from 10 to 0.000001 ng. Each DNA template was amplified by HDA using sterile distilled water and sterile platelet product as negative controls. The HDA products were analysed by SYBR Green I and 2% agarose gel electrophoresis. The experiments were performed in triplicate.

### Cross-reaction testing

The specificity of HDA/SYBR Green I was evaluated with DNA extracted from microbial pathogens other than bacteria. These pathogens included the common fungi *Candida albicans*, *Cryptococcus neoformans*, *Aspergillus* spp., *Rhizopus* spp. (kindly provided with no patient data links by the Microbiology Unit, Faculty of Medicine Ramathibodi Hospital, Mahidol University, Bangkok, Thailand), and bloodborne hepatitis B virus (kindly provided with no patient data links by Prof. Yong Poovorawan, the Centre of Excellence in Clinical Virology, Faculty of Medicine, Chulalongkorn University, Bangkok, Thailand. Human genomic DNA was extracted from a sterile discarded platelet product and included in cross-reaction testing. A total of 10 ng of individual DNA templates was used for HDA amplification. The HDA product was evaluated by SYBR Green I and 2% agarose gel electrophoresis. Sterile distilled water and *E. coli* colony DNA were used as negative and positive controls, respectively. The experiments were performed in triplicate.

### Test performance analyses

The sensitivity and specificity of HDA/SYBR Green I were evaluated with the FDA-recommended BACT/ALERT system. Sensitivity (a true positive rate) was computed from sensitivity (%) = TP/(TP + FN) × 100, where ‘TP’ is the number of positive samples, and ‘FN’ is the number of false negatives when spiked samples were used. Specificity (a true negative rate) was computed from specificity (%) = TN/(TN + FP) × 100, where ‘TN’ is the number of negative samples and ‘FP’ is the number of false-positives when nonspiked samples (sterile distilled water and sterile platelet product) were used. To further evaluate the reliability of HDA/SYBR in detecting bacterial contamination in platelet products, the percent agreement between the two methods (HDA/SYBR Green I and BacT/Alert® system) was calculated. The Kappa result was interpreted as follows: values ≤ 0 indicated no agreement, 0.01–0.20 indicated slight agreement, 0.21–0.40 indicated fair agreement, 0.41–0.60 indicated moderate agreement, 0.61–0.80 indicated substantial agreement, and 0.81–1.00 indicated almost perfect agreement.

## Results

### Establishment of spiked platelet product samples

The average bacterial concentration in the platelet products from Day 0 to Day 5 of storage is shown in Table 1. A visible colony of every bacterial strain, except *S. epidermidis*, was initially observed in platelet products collected from Day 1 of storage, with concentrations ranging from 8.8 × 10^2^ to 1.9 × 10^3^ CFU/ml. In contrast, the colony of *S. epidermidis* was slower on Day 2 of storage, with a concentration of 2.2 × 10^3^ CFU/ml. The number of bacterial cells in platelet products increased during a more extended storage period in a time-dependent manner. The maximum bacterial concentration was 1.2 × 10^10^ CFU/ml, which was found on Day 5 of storage when inoculated with *S. marcescens*.

**Table 1:** Detection of bacterial contaminants in platelet products using HDA/SYBR Green I assay compared with colony count during storage periods.

Bacterial strains	Storage periods of platelet products					
	Day 0	Day 1	Day 2	Day 3	Day 4	Day 5
*S. aureus* (ATCC29523)	0/0^a^ No visible colony	0/0 (1.7 × 10^3^ CFU/ml)^b^	5/5 (1.3 × 10^8^ CFU/ml)	5/5 (5.0 × 10^8^ CFU/ml)	5/5 (6.6 × 10^8^ CFU/ml)	5/5 (1.4 × 10^9^ CFU/ml)
*S. epidermidis*	0/0 No visible colony	0/0 (0 CFU/ml)	4/5 (2.2 × 10^3^ CFU/ml)	5/5 (8.4 × 10^5^ CFU/ml)	5/5 (9.5 × 10^6^ CFU/ml)	5/5 (1.2 × 10^8^ CFU/ml)
*B. cereus*	0/0 No visible colony	4/5 (1.9 × 10^3^ CFU/ml)	5/5 (2.0 × 10^8^ CFU/ml)	5/5 (4.1 × 10^8^ CFU/ml)	5/5 (6.9 × 10^8^ CFU/ml)	5/5 (7.8 × 10^8^ CFU/ml)
*E. coli* (ATCC25922)	0/0 No visible colony	1/5 (1.8 × 10^3^ CFU/ml)	5/5 (1.1 × 10^8^ CFU/ml)	5/5 (2.4 × 10^8^ CFU/ml)	5/5 (2.2 × 10^8^ CFU/ml)	5/5 (2.9 × 10^9^ CFU/ml)
*S. marcescens*	0/0 No visible colony	1/5 (8.8 × 10^2^ CFU/ml)	4/5 (7.4 × 10^4^ CFU/ml)	5/5 (2.6 × 10^9^ CFU/ml)	5/5 (1.1 × 10^10^ CFU/ml)	5/5 (1.2 × 10^10^ CFU/ml)

^a^Number of a positive HDA/SYBR Green I reactions/replicates.

^b^Bacterial concentration acquired by colony count assay.

### BacT/ALERT® culture system detected bacterial contamination in platelet products

The BacT/ALERT® culture system could detect bacterial contamination in all storage periods of spiked platelet products, starting from the collection day (Day 0). The average processing time of the BacT/ALERT® system from sample incubation until a positive signal was detected ranged from 10 h and 54 min to 18 h and 15 min (Supplement Table 1).

### Detection of bacterial contamination by HDA/SYBR green I

**Figure 2:**
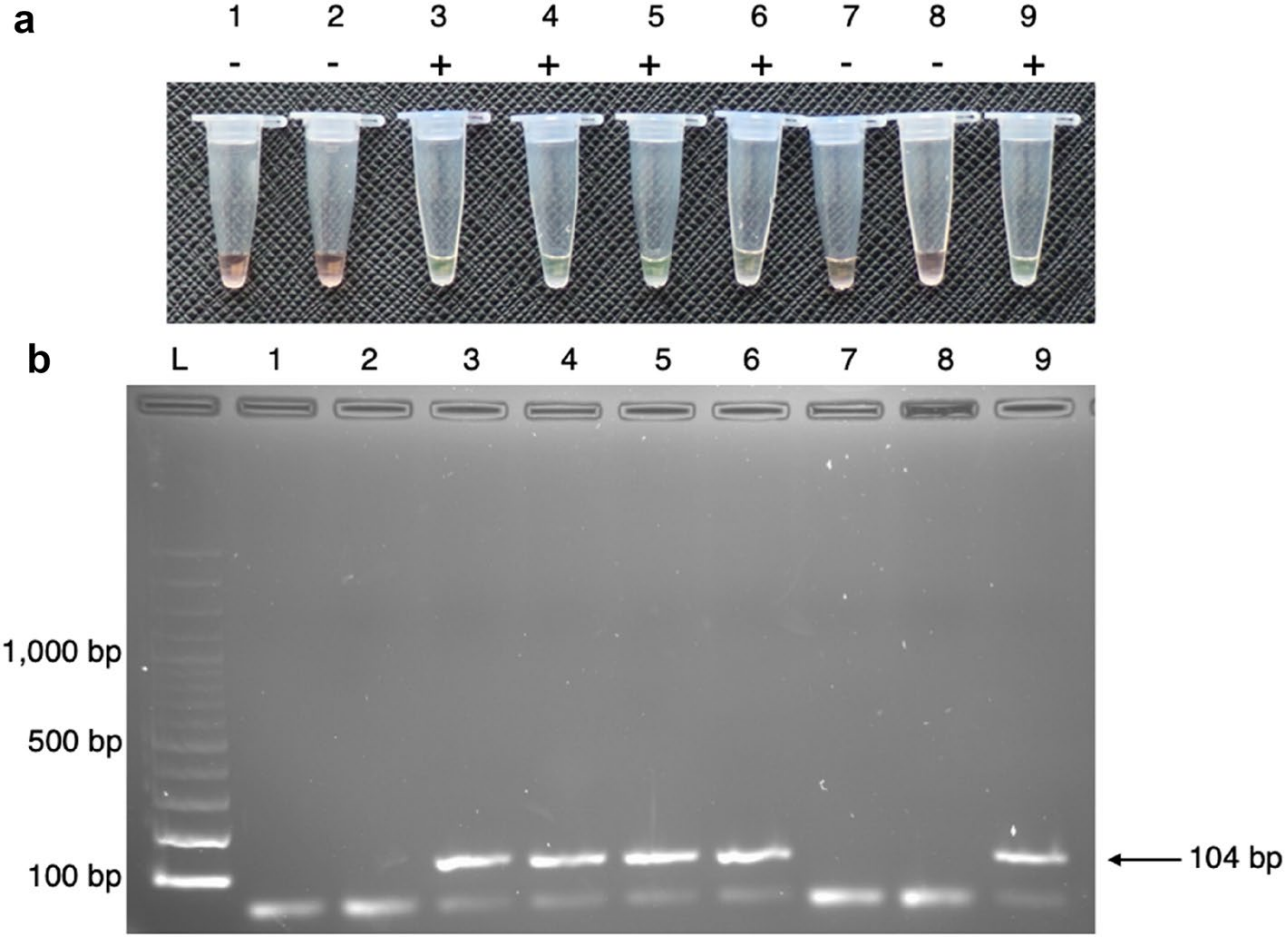
HDA/SYBR Green I detected bacterial contaminants in platelet products. Samples 1 to 6 are DNA samples from Day 0 to Day 5, respectively, of platelet products containing spiked *E. coli*. Samples 7 and 8 are negative controls (sterile distilled water and sterile platelet product, respectively). Sample 9 is a positive control (*E. coli* colony DNA). (**a**) HDA products were naked eye detected by SYBR green I directly in the reaction tubes. Tubes 1, 2, 7, and 8 show an orange colour, which implies a negative amplification result. Tubes 3, 4, 5, 6, and 9 show a green colour, which indicates a positive amplification result. Documented by Canon EOS 760D: ISO 800, aperture f/3.5, and speed shutter 1/125. (**b**) Cropped gel image demonstrates HDA products that were detected by 2% agarose gel electrophoresis (documented by Gel Doc™ XR ChemiDoc^TM^XRS). Lane L: 100-bp DNA ladder. Reactions in lanes 1, 2, 7, and 8 show no visible target band. Reactions in lanes 3, 4, 5, 6, and 9 show an amplification product size of 104 bp. Original images are presented in Supplementary Fig. 2.

After complete optimisation of HDA/SYBR Green I, the appropriate assay conditions were achieved. When evaluated with genomic DNA derived from a colony of 5 representative bacterial strains, SYBR Green I stayed orange in a negative control tube due to the lack of amplification of the HDA product. In contrast, SYBR Green I switched to shining green in the positive control tubes, implying that DNA products were amplified by HDA with 16S rRNA gene-specific primers (Fig. 2). HDA/SYBR Green I concordantly exhibited positive and negative results for agarose gel electrophoresis. The presence of a bottom primer-dimer band on an agarose gel in Fig. 2 (Lanes 1, 2, 7 and 8) did not cause a false-positive or background on SYBR Green I detection or interfere with the assay. When testing with Day 0 to Day 5 of stored (contaminated samples) and sterile platelet products (sterile samples) in 5 replicates, HDA/SYBR Green I could correctly detect positive and negative samples, as described in Table 1. Platelets inoculated with *B. cereus*, *E. coli,* and *S. marcescens *were first detected on Day 1 of storage (average bacterial concentration was 1.9 × 10^3^, 1.8 × 10^3^, and 8.8 × 10^2^ CFU/ml, respectively), whereas almost of the rest of the contaminants started to be detected on Day 2 of storage (bacterial concentration ranged from 7.4 × 10^4^ to 2.0 × 10^8^ CFU/ml). The average test time of HDA/SYBR Green I, from the reaction began until the sample readout, was 1 h and 40 min.

### Performance of contaminated bacterial detection by HDA/SYBR Green I

The performance of HDA/SYBR Green I to detect bacterial contamination in platelet products was validated with the BacT/ALERT® culture system. HDA/SYBR Green I could not detect bacterial contamination in freshly prepared Day 0 of storage samples and exhibited a very low sensitivity when tested with Day 1 of storage samples. The sensitivity and specificity of HDA/SYBR Green I when tested with Day 1 of storage samples was 4.00% and 100%, respectively, with slight agreement with a standard method. However, when tested with samples from Day 2 of storage or later, HDA/SYBR Green I showed markedly increased sensitivity of 98.67–100% and specificity of 100% with almost perfect agreement (Table 2).

**Table 2:** Performance of HDA/SYBR Green I assay validated by BACT/ALERT culture system during storage periods.

Storage periods of platelet products	Sensitivity	Specificity	PPV^a^	NPV^b^	Cohen kappa's coefficient
Day 0	0.00% (95% CI: 0.00–2.46%)	100% (95% CI: 94.22–100%)	– –	29.52% (95% CI: 29.52–29.52%)	0.000 (95% CI: 0.000–0.000)
Day 1	4.00% (95% CI: 1.48–8.50%)	100% (95% CI: 94.04–100%)	100% –	29.41% (95% CI: 28.74–30.09%)	0.023 (95% CI: 0.004–0.042)
Day 2	98.67% (95% CI: 95.27–99.84%)	100% (95% CI: 94.04–100%)	100% –	96.77% (95% CI: 88.33–99.17%)	0.977 (95% CI: 0.945–1.000)
Day 3	100% (95% CI: 97.57–100%)	100% (95% CI: 94.04–100%)	100% –	100% –	1.000 (95% CI: 1.000–1.000)
Day 4	100% (95% CI: 97.57–100%)	100% (95% CI: 94.04–100%)	100% –	100% –	1.000 (95% CI: 1.000–1.000)
Day 5	100% (95% CI: 97.57–100%)	100% (95% CI: 94.04–100%)	100% –	100% –	1.000 (95% CI: 1.000–1.000)

^a^Positive predictive values.

^b^Negative predictive values.

### LOD and cross-reactivity of HDA/SYBR green I

**Figure 3:**
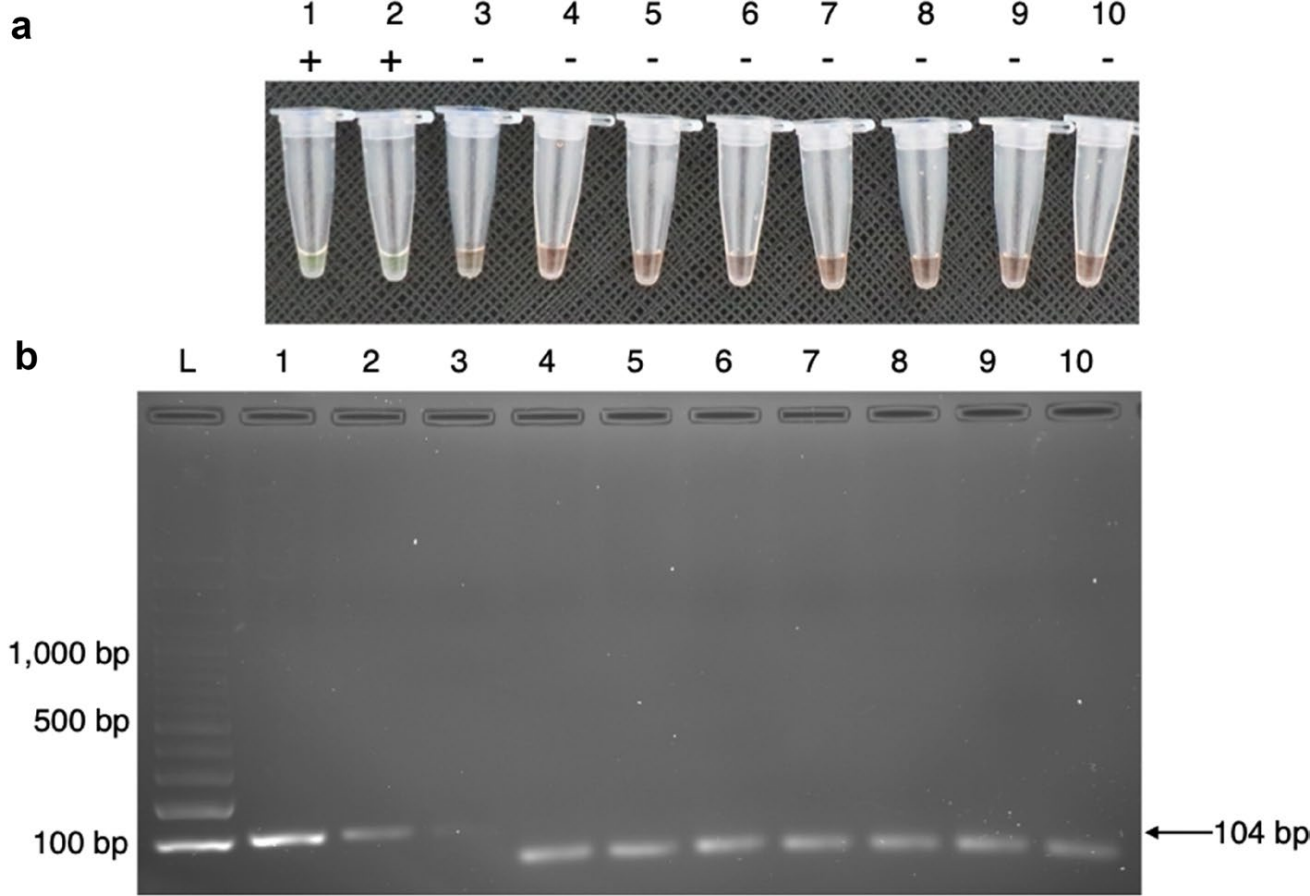
LOD of HDA/SYBR Green I. *S. marcescens* colony DNA was tenfold serially diluted from 10 to 0.000001 ng (samples 1 to 8, respectively). Samples 9 and 10 are negative controls (sterile distilled water and sterile platelet product, respectively). (**a**) HDA products were detected by SYBR Green I. Tubes 1 and 2 show a green colour, which indicates a positive amplification result. Tubes 3 to 10 shows an orange colour, which implies a negative amplification result. Documented by Canon EOS 760D: ISO 800, aperture f/4.5, and speed shutter 1/125. (**b**) Cropped gel image demonstrates HDA products that were detected by 2% agarose gel electrophoresis (documented by Gel Doc™ XR ChemiDoc^TM^XRS). Lane L: 100-bp DNA ladder. Reactions in lanes 1, 2, and 3 show an amplification product size of 104 bp. Reactions in lanes 4 to 10 show no visible target band. Original images are presented in Supplementary Fig. 3.

**Figure 4:**
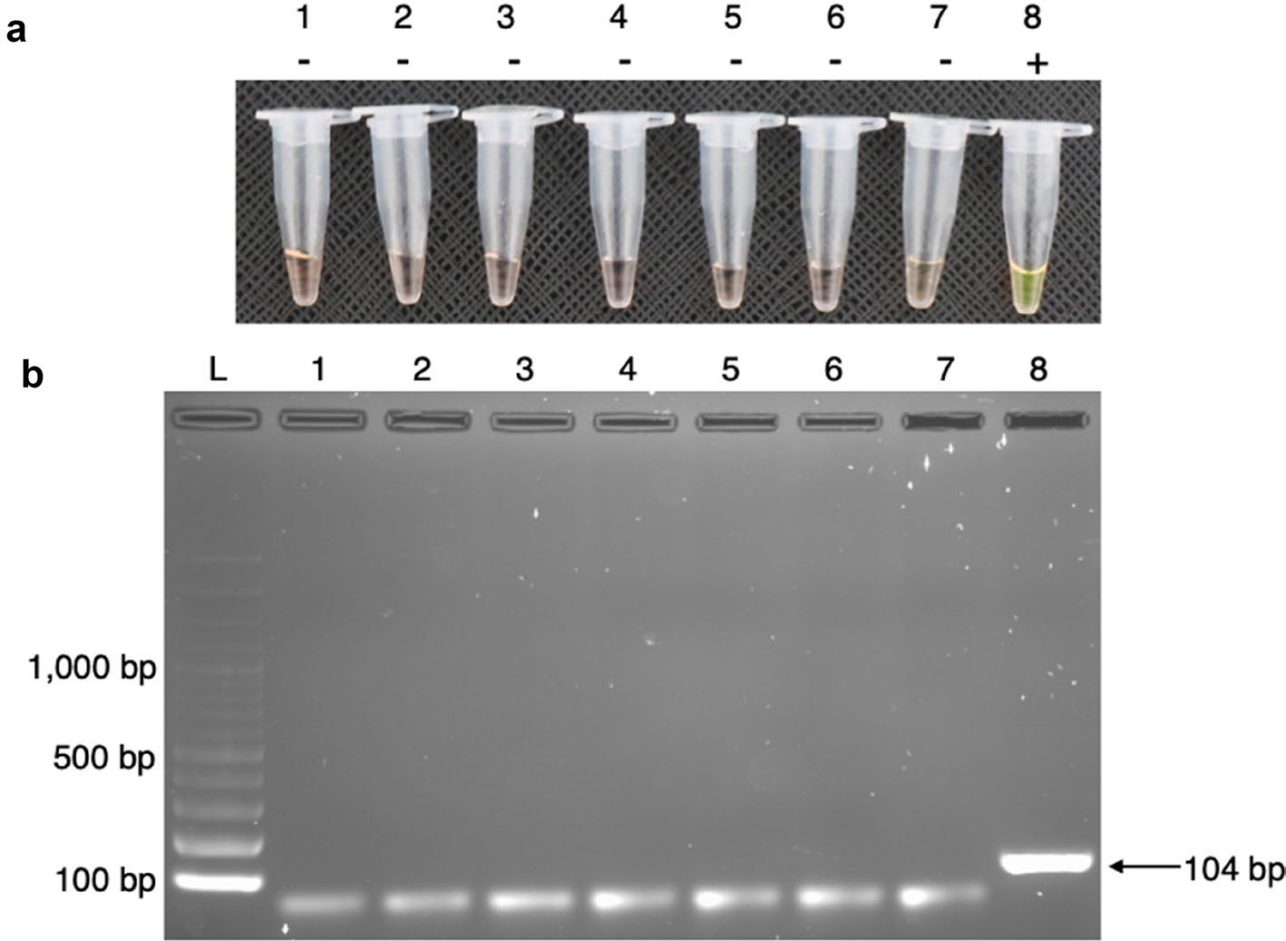
Cross-reactivity testing of HDA/SYBR Green I. (**a**) Tubes 1 to 6 are DNA from *C. albicans*, *C. neoformans*, *Aspergillus* spp., *Rhizopus* spp., hepatitis B virus and human genomic DNA, respectively. Tube 7 is a negative control, showing an orange colour, which indicates no amplification. Tube 8 is a positive control (*E. coli* colony DNA), showing a green colour, which indicates a positive amplification result. Documented by Canon EOS 760D: ISO 1600, aperture f/4.5, and speed shutter 1/20. (**b**) Cropped gel image demonstrates HDA products that were detected by 2% agarose gel electrophoresis (documented by Gel Doc™ XR ChemiDoc^TM^XRS). Lane L: 100-bp DNA ladder. No visible target band is seen in lanes 1–7, indicating a negative amplification result. An amplification product size of 104 bp was seen in lane 8, indicating a positive amplification result. Original images are presented in Supplementary Fig. 4.

The LOD of the HDA/SYBR Green I assay was 1 ng. When the HDA product was detected by agarose gel electrophoresis, the LOD was as low as 0.1 ng. HDA/SYBR Green I did not cross-react with *C. albicans*, *C. neoformans*, *Aspergillus* spp., *Rhizopus* spp., hepatitis B virus, or human genomic DNA. Figures 3 and 4 show the LOD and cross-reactivity testing of HDA/SYBR Green I, respectively.

## Discussion

A transfusion with bacterial contaminated platelets can lead to transfusion-transmitted diseases such as transfusion-transmitted bacterial infection (TTBI) and septic transfusion reactions (STRs), which are significant sources of morbidity and mortality. Between 1 in 1000 to 2000 platelet units are bacterially contaminated. Thus, a strategy to relieve the risk of bacterial contamination in platelet products is challenging. Different strategies have been proposed, including preventing bacterial contamination during platelet collection, pathogen reduction/inactivation technologies and testing methods for platelet bacterial contamination^11^. In 2004, AABB standard 5.1.5.1 instructed that the blood bank or transfusion service should have methods to limit and detect bacterial contamination in platelet products before applying to the patients to minimise any adverse reactions^4^.

The BacT/ALERT® culture system is the primary automated culture-based method commonly used worldwide and in major transfusion service centres in Thailand. The assay has been approved by the FDA and is regarded as the gold standard for bacterial contaminant screening in platelet products after collection. In our experimental setting, the BacT/ALERT® culture system effectively detected bacterial contamination in collection day (Day 0) and early storage (Day 1) platelet samples inoculated with bacterial strains (e.g., *S. aureus*, *S. epidermidis*, *B. cereus*, *E. coli*, and *S. marcescens*) with an incubation time of 10–18 h. A previous study showed that the level of detection of the BacT/ALERT® culture system against those relative bacteria was as low as 1–10 CFU/ml^5^. The BacT/ALERT® system has been shown to reduce the incidence of TTBI in screening programs for bacterial contamination of platelet products^12^. However, this current automatic culture system may not effectively detect biofilm-producing organisms^13^. Moreover, due to high cost-effectiveness, implementing the BacT/ALERT® system may be limited in many transfusion service routines in Thailand. Currently, molecular biology techniques, such as real-time PCR, are being developed to detect bacterial contamination in platelet products targeting highly conserved 16S rRNA and 23S rRNA genes^8,14^. The analytical sensitivities ranged from 5 to 50 CFU/ml.

However, the ideal screening test should have a high diagnostic sensitivity and specificity and be affordable, reliable, and rapid. Real-time PCR relies on a specific thermocycler, which is not yet widely used in transfusion service routines to detect bacterial platelet contamination. Isothermal-based amplification is a molecular method that depends on a constant temperature during the entire amplification process. Over 15 types of isothermal amplification techniques have been described. However, some techniques have yet to be developed beyond proof of concept or academic research. The most representative and applied isothermal amplification methods, such as loop-mediated amplification (LAMP), recombinase polymerase amplification (RPA), helicase-dependent amplification (HDA), rolling circle amplification (RCA), strand displacement amplification (SDA), nucleic acid sequence-based amplification (NASBA). LAMP has been the most widely used isothermal amplification method, which relies on a *Bst* DNA polymerase and a set of 4–6 primers for amplification. LAMP is extremely sensitive and has high specificity with an amplification time of less than 1 h at 60–65 °C. Even LAMP is simple to operate, but a higher risk of contamination and complexity in primer design is a disadvantage^15^. In our pilot study, we focused on an isothermal amplification technique that is simple either in a primer design or an operation step. RPA was attempted in the first period of our development. RPA offers many advantages over other isothermal amplification techniques, such as a simple primer design, a high speed and sensitivity, a broad temperature range (25–45 °C), and multiplexing availability. However, TwistAmp® RPA kits contain a small amount of *E. coli* DNA due to the manufacturing method^16^, leading to a false positive in our pilot study (data not shown). According to the manufacturer's instruction manual, RPA reactions are unsuitable for developing diagnostics for *E. coli* if sequence homology with strains K12 and BL21 is present^16^. *E. coli* is one of the most contaminants in platelet products; thus, RPA was considered unsuitable for our developed assay in this study. HDA is one of the most straightforward isothermal amplification schemes^7^ requiring only 2 primers per target and a double-strand DNA as a template^15^. HDA is compatible with different detection platforms, including fluorescent DNA intercalator and lateral flow device, which is very practicable to use in general laboratories. Our developed HDA/SYBR Green I assay has an affordable price (~ 7 USD per reaction). However, the assay has a slightly higher cost than LAMP (~ 2 USD per reaction) and RPA (~ 5 USD per reaction). HDA has been successfully developed as a novel molecular-based method for the rapid and sensitive detection of bacteria (e.g.,* E. coli*,* S. aureus*, *Chlamydia trachomatis* and *Neisseria gonorrhoeae*) and viruses (e.g., human papillomavirus 16 and 18)^6^. The optimum HDA temperature ranges from 60 to 65 °C; thus, the entire reaction can be constantly operated by a simple heating block that is generally used in blood bank laboratories.

Here, HDA was chosen to amplify bacterial target DNA, the 16S rRNA gene, to establish “advanced instrument-free” amplification that depends on a simple heat generating box available in general transfusion service centres. The 16S rRNA gene has been a mainstay of molecular-based bacterial investigation for decades and can be implemented in aerobic, anaerobic, facultative, and fastidious bacteria. The copy numbers of 16S rRNA are diverse among bacterial phyla. They were high in Firmicutes, Gammaproteobacteria and Fusobacteria, with averages greater than five copies per genome. In contrast, most bacterial phyla contain at least a single 16S rRNA copy^17^. Target selection and primer design are critical in assay development. Therefore, the target sequence and initial primer selection were first verified in silico before the candidate set of primers was experimentally screened with reference strains. However, our 16S rRNA primers were designed in silico to cover 275,057 eubacterial species, including the commonly contaminating bacteria, and the detection of some species may eventually fail^10^.

The HDA/SYBR Green I assay provided high sensitivity and specificity and was rapid for detecting bacterial contamination in platelet products by the naked-eye. The developed assay was conducted under a constant temperature of 65 °C, and the test time was approximately 1 h and 40 min from sampling to readout. In our study, the early sampling with a small sample volume (1 ml) might have caused false negative results of the HDA/SYBR Green I assay compared with the BacT/ALERT® system, which was processed with a larger sample volume (10 ml). In a previous study, a high-volume extraction (2.4 ml of sample volume) of total DNA by magnetic bead technology was shown to enhance the sensitivity of the 23S rRNA real-time reverse transcription PCR assay for detecting bacterial contamination of platelet products^14^. HDA/SYBR Green I showed poor sensitivity in detecting bacterial contamination in samples collected from early shelf life, Day 0 and Day 1. However, the assay showed an enhanced yield of detection when performed with medium to late shelf life, Day 2 of storage or later platelet products, with a sensitivity ranging from 98.67 to 100%. The sample collection at an early stage of platelet shelf life can cause a sampling error, owing to a low bacterial contamination number at the start time of storage, mainly when slow-growing bacteria are implicated in the contamination. Previously, the risk of sampling errors continuously decreased during storage; the detection rate of bacterial contamination changed from 14.9% for sampling on Day 1–100% for sampling on Day 7^18^. The bacterial load in platelet products has been suggested to be associated with storage times. One approach to improving the sensitivity of contaminated platelet screening could be to collect samples later in platelet storage (e.g., Day 3–5 of storage)^9^. Another factor that might interfere with the efficacy of the HDA assay during the amplification process is the presence of human genomic DNA in clinical samples, presumably through binding HDA enzymes. Doseeva et al. showed that more than 200 ng of human genomic DNA per HDA assay significantly interfered with the specific amplification^19^. Removal of background human genomic DNA originating from platelet cells may increase either sensitivity or specificity of the HDA assay.

Our study has proven a concept to combine isothermal-based amplification, HDA, with naked-eye detection using SYBR Green I to detect bacterial contamination in platelet products. HDA/SYBR Green I is less laborious and expensive (equipment, reagents) than real-time PCR and the BacT/ALERT® system. The total test time of HDA/SYBR Green I is faster than that of culture-based assays, and can be implemented for routine contaminant bacterial screening in platelet products before providing them to a patient. The ideal test to detect bacterial contamination in platelet products should be highly sensitive (1 CFU/ml), specific, rapid (less than 2 h), inexpensive, reliable, and simple^20^. HDA/SYBR Green I first detected bacteria on Day 1 of storage (1.9 × 10^3^ CFU/ml) as the earliest detection, whereas almost of the rest of the contaminants started to be detected on Day 2 of storage (7.4 × 10^4^ to 2.0 × 10^8^ CFU/ml). The analytical sensitivity of HDA/SYBR Green I was similar to that obtained from real-time PCR for *S. epidermidis* (2.9 × 10^4^ CFU/ml) and *E. coli* (2.2 × 10^4^ CFU/ml) in a previous study^14^. Earlier published surveillance data show that platelet units with bacterial concentrations of < 10^5^ CFU/ml are much less likely to cause severe STRs than units with higher bacterial concentrations^21^. HDA/SYBR Green I potentially detected bacterial concentrations in platelet products at the minimum clinical significance cut-off of < 10^5^ CFU/ml. Nevertheless, the sensitivity of our HDA/SYBR Green I assay requires further improvement.

## Conclusion

In conclusion, the HDA/SYBR Green I assay in combination with late sampling can enhance the possibility of detecting bacteria in platelet products and can be easily adopted in a blood bank service for the routine screening of bacterial contaminants. Our developed HDA/SYBR Green I assay is rapid and simplistic and only requires an easy-to-find heat box, which is available in general blood bank laboratories, for the amplification step. This technique is suitable for further development as an alternative method to detect bacterial contamination in platelet products in the future.

## Supplementary Information

Supplementary Information 1.

Supplementary Information 2.

Supplementary Information 3.

Supplementary Information 4.

Supplementary Information 5.

Supplementary Information 6.

Supplementary Information 7.

Supplementary Information 8.

Supplementary Information 9.

## Data Availability

The datasets analysed during the current study are available in the DNA Data Bank of Japan (DDBJ) repository, LC730901, LC730902, LC730903, LC730904, and LC746965 for analysed sequences of 16S rRNA of *S. aureus*, *S. epidermidis*, *B. cereus*, *E. coli* and *S. marcescens*, respectively, generated in the study.
