# Supplementary figures and images for: Implementation of helicase-dependent amplification with SYBR Green I for prompt naked-eye detection of bacterial contaminants in platelet products
Source: Sci Rep. 2023 Feb 24;13:3238. doi: 10.1038/s41598-023-30410-8 (PMC9958189; doi:10.1038/s41598-023-30410-8)

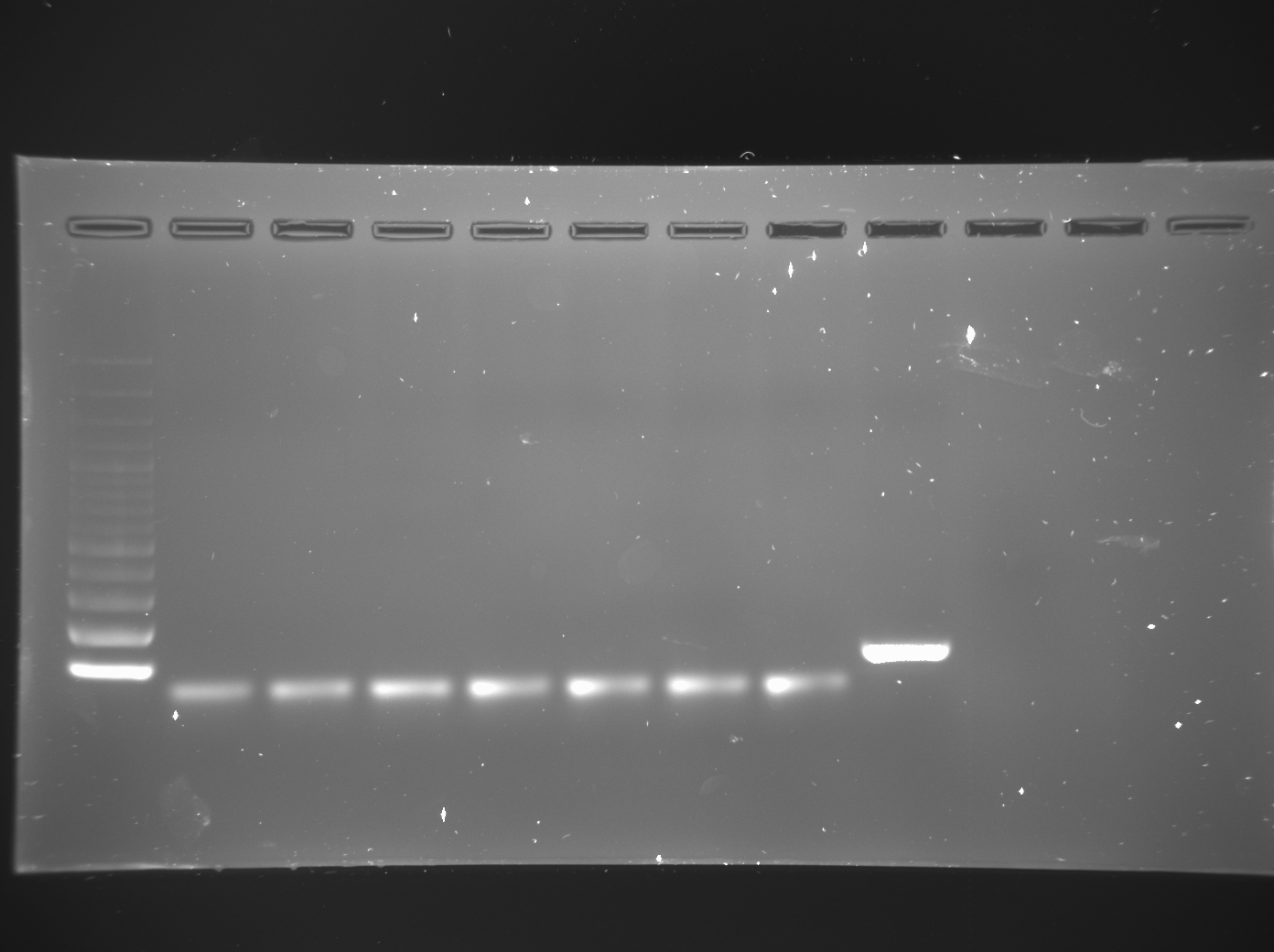

Supplement: Supplementary file 1 — Supplementary Information 1. [file 41598_2023_30410_MOESM1_ESM.tif]

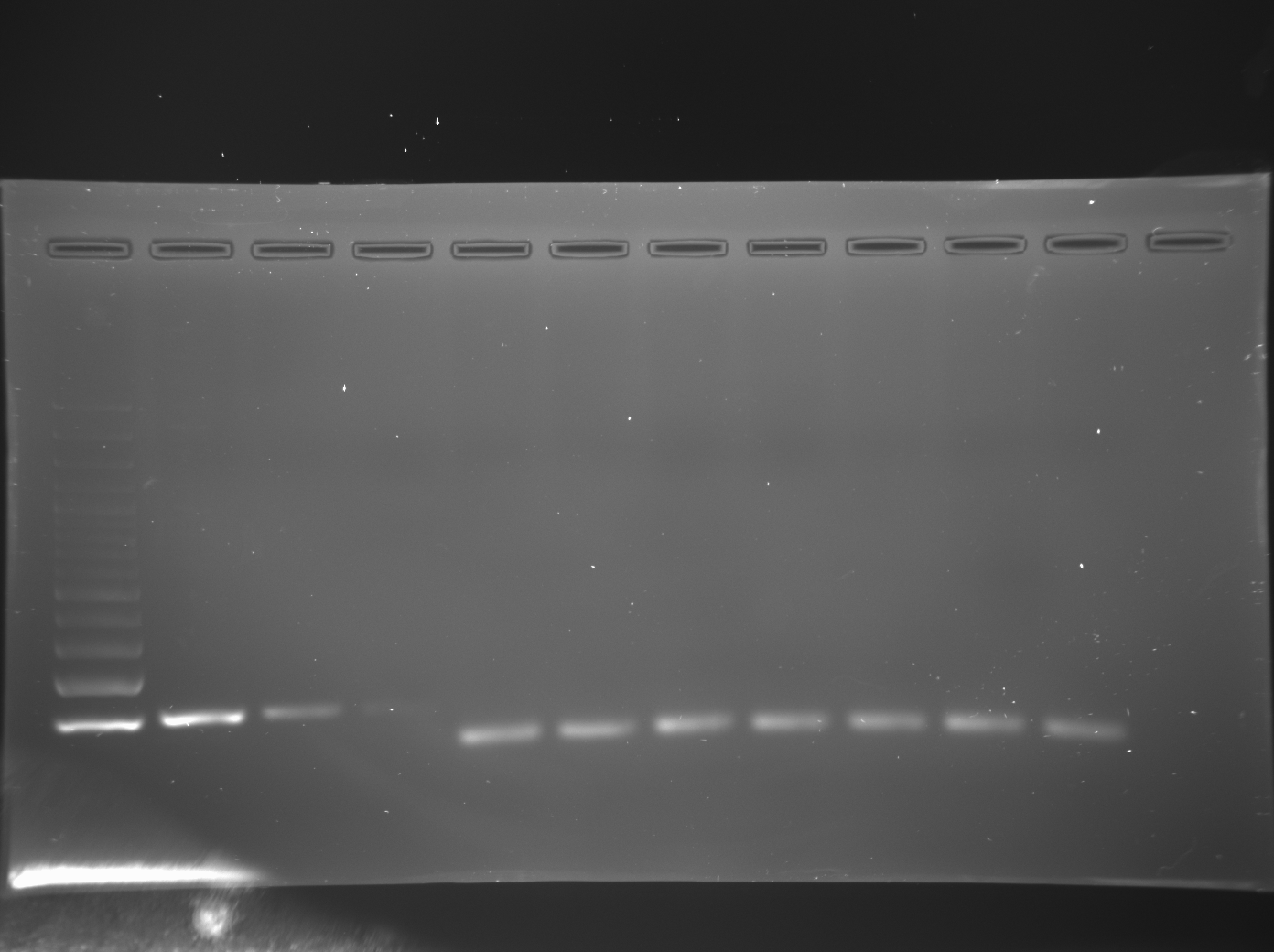

Supplement: Supplementary file 4 — Supplementary Information 4. [file 41598_2023_30410_MOESM4_ESM.tif]

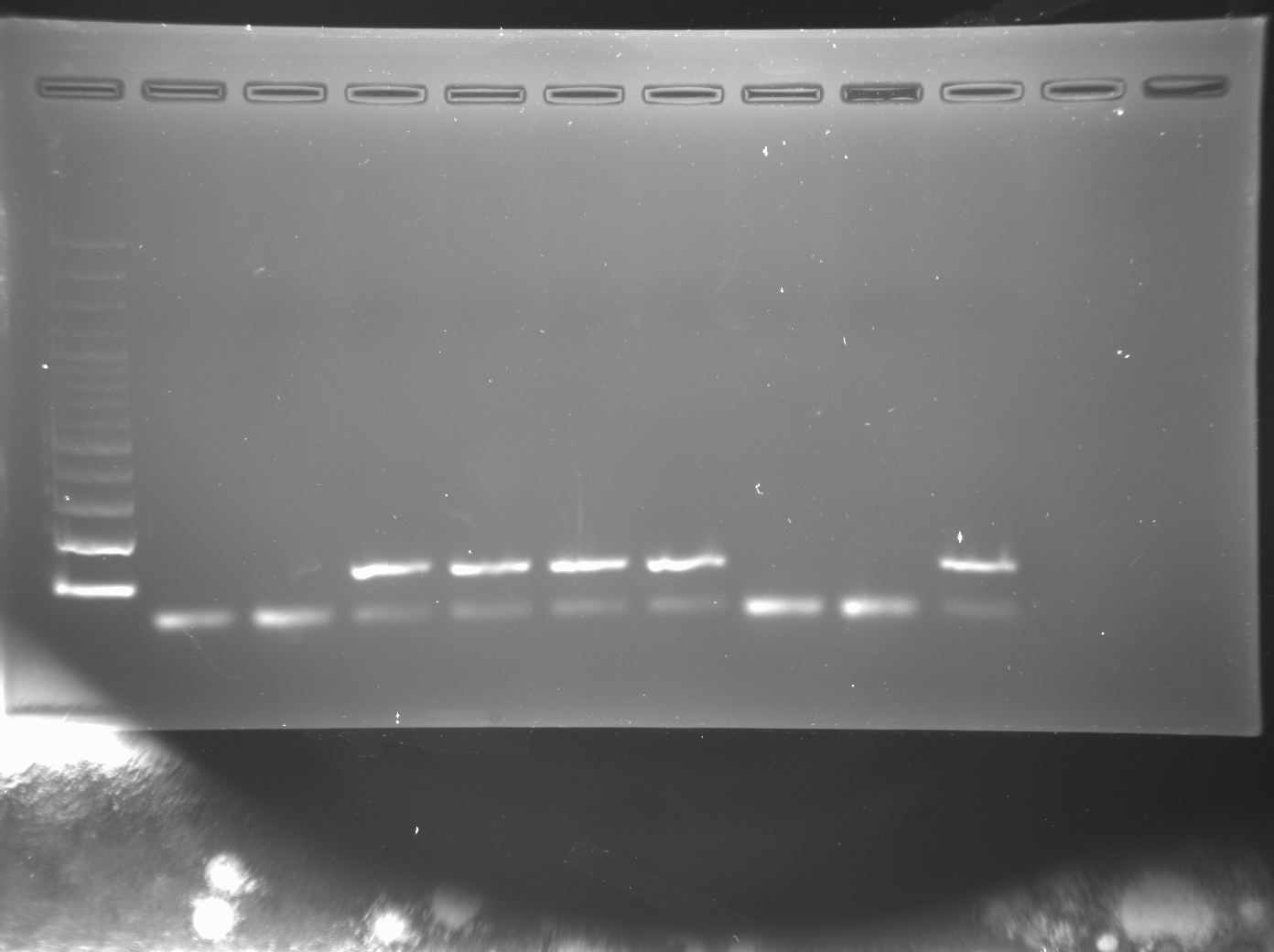

Supplement: Supplementary file 5 — Supplementary Information 5. [file 41598_2023_30410_MOESM5_ESM.tif]

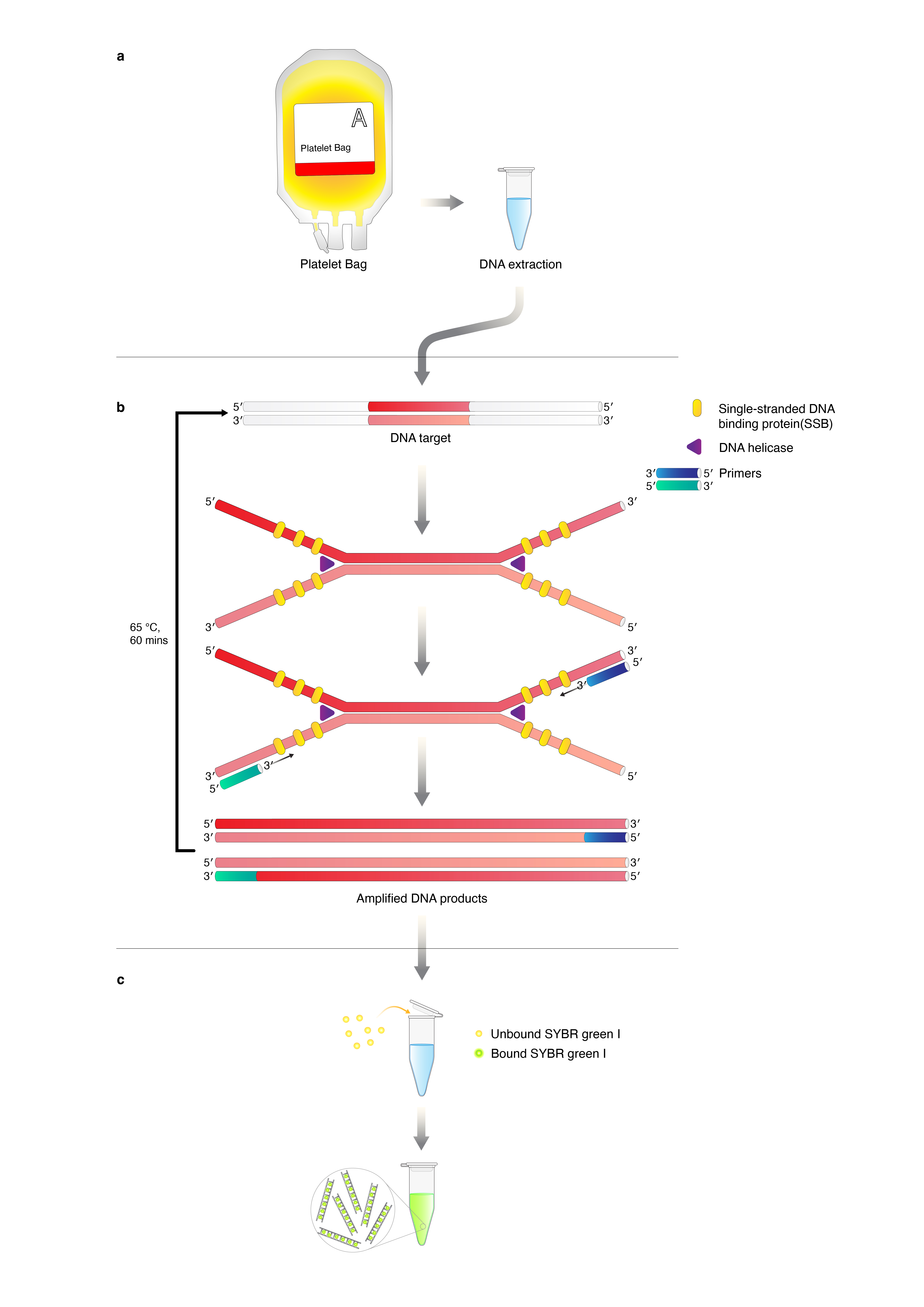

Supplement: Supplementary file 6 — Supplementary Information 6. [file 41598_2023_30410_MOESM6_ESM.jpg]
