# Supplementary material for: Implementation of helicase-dependent amplification with SYBR Green I for prompt naked-eye detection of bacterial contaminants in platelet products
Source: Sci Rep. 2023 Feb 24;13:3238. doi: 10.1038/s41598-023-30410-8 (PMC9958189; doi:10.1038/s41598-023-30410-8)
Supplement: Supplementary file 3 — Supplementary Information 3. [file 41598_2023_30410_MOESM3_ESM.pdf]

Supplement table 1 Detection of bacterial contaminants in platelet products using BacT/ALERT® culture system compared with colony count during storage periods.

| Bacterial strains | Storage periods of platelet products | | | | | |
|---|---|---|---|---|---|---|
| | Day 0 | Day 1 | Day 2 | Day 3 | Day 4 | Day 5 |
| *S. aureus* (ATCC29523) | 5/5 [a] No visible colony | 5/5 (1.7 x$10^3$ CFU/ml) [b] | 5/5 (1.3x$10^8$ CFU/ml) | 5/5 (5.0x$10^8$ CFU/ml) | 5/5 (6.6x$10^8$ CFU/ml) | 5/5 (1.4x$10^9$ CFU/ml) |
| *S. epidermidis* | 5/5 No visible colony | 5/5 (0 CFU/ml) | 5/5 (2.2x$10^3$ CFU/ml) | 5/5 (8.4x$10^5$ CFU/ml) | 5/5 (9.5x$10^6$ CFU/ml) | 5/5 (1.2x$10^8$ CFU/ml) |
| *B. cereus* | 3/5 No visible colony | 5/5 (1.9 x$10^3$ CFU/ml) | 5/5 (2.0x$10^8$ CFU/ml) | 5/5 (4.1x$10^8$ CFU/ml) | 5/5 (6.9x$10^8$ CFU/ml) | 5/5 (7.8x$10^8$ CFU/ml) |
| *E. coli* (ATCC25922) | 5/5 No visible colony | 5/5 (1.8 x$10^3$ CFU/ml) | 5/5 (1.1x$10^8$ CFU/ml) | 5/5 (2.4x$10^8$ CFU/ml) | 5/5 (2.2x$10^8$ CFU/ml) | 5/5 (2.9x$10^9$ CFU/ml) |
| *S. marcescens* | 5/5 No visible colony | 5/5 (8.8 x$10^2$ CFU/ml) | 5/5 (7.4x$10^4$ CFU/ml) | 5/5 (2.6x$10^9$ CFU/ml) | 5/5 (1.1x$10^{10}$ CFU/ml) | 5/5 (1.2x$10^{10}$ CFU/ml) |

[a] Number of positive signal detections by BacT/ALERT® culture system/replicates
[b] Bacterial concentration acquired by colony count assay
